# Supplementary material for: The Relationship between Nutrient Intake and Cataracts in the Older Adult Population of Korea
Source: Nutrients. 2022 Nov 23;14(23):4962. doi: 10.3390/nu14234962 (PMC9739297; doi:10.3390/nu14234962)
Supplement: Supplementary file 1 [file nutrients-14-04962-s001.zip › nutrients-1980738-supplementary.pdf]

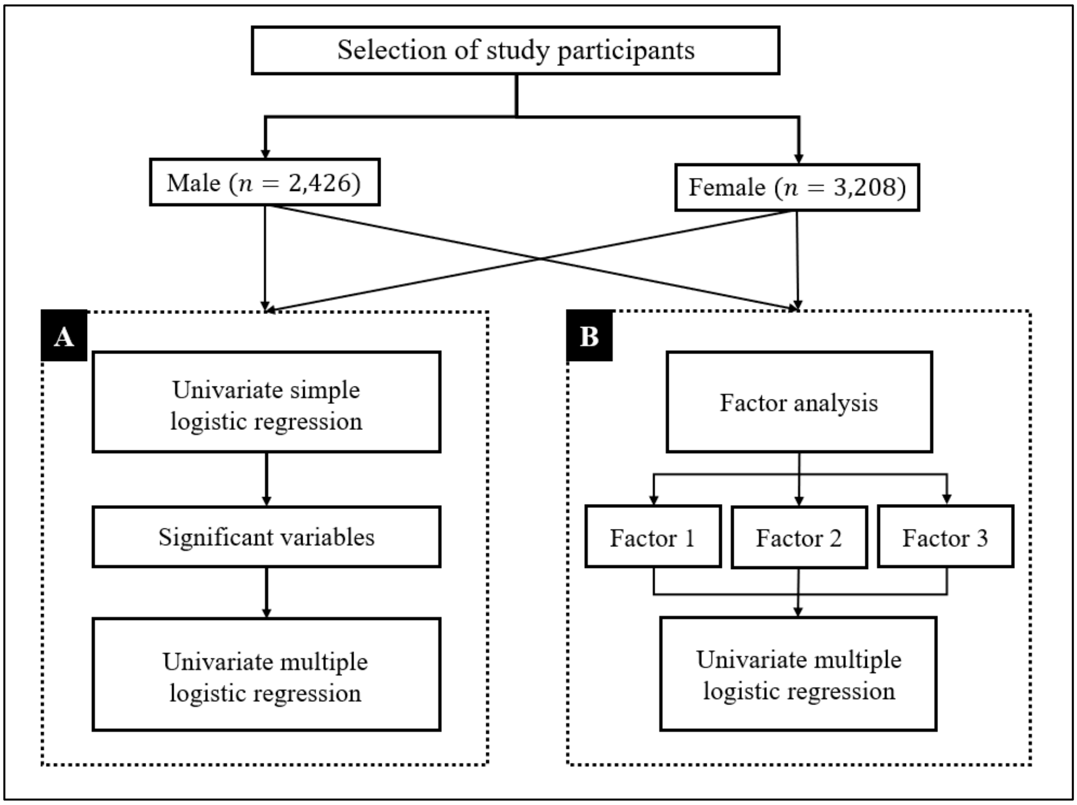

Figure S1. Overall statistical analysis method (A) For male and female, significant variables were extracted through univariate simple logistic regression. Then, univariate multiple logistic regression was performed using extracted variables. (B) For male and female, three factors were extracted from independent variables by factor analysis. Then, we performed univariate multiple logistic regression using extracted factors.

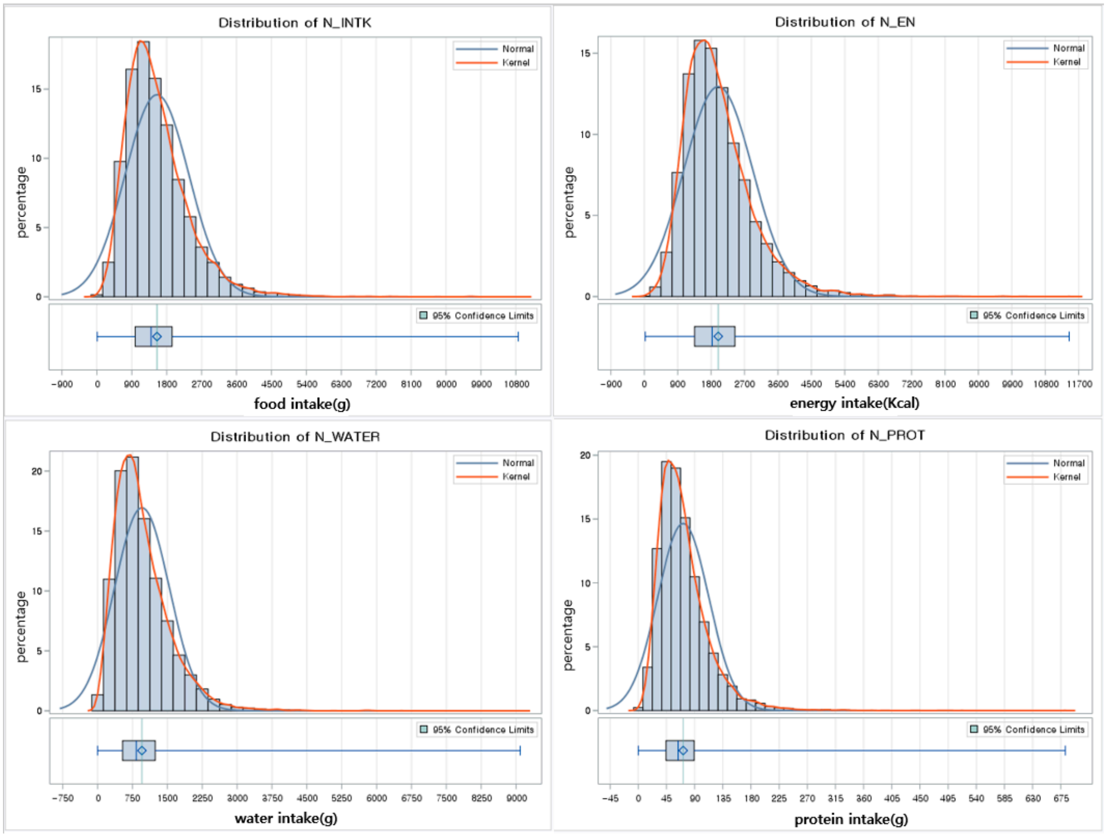
Distribution of N_INTK
Normal
Kernel
95% Confidence Limits
percentage
food intake(g)
Distribution of N_EN
Normal
Kernel
95% Confidence Limits
percentage
energy intake(Kcal)
Distribution of N_WATER
Normal
Kernel
95% Confidence Limits
percentage
water intake(g)
Distribution of N_PROT
Normal
Kernel
95% Confidence Limits
percentage
protein intake(g)

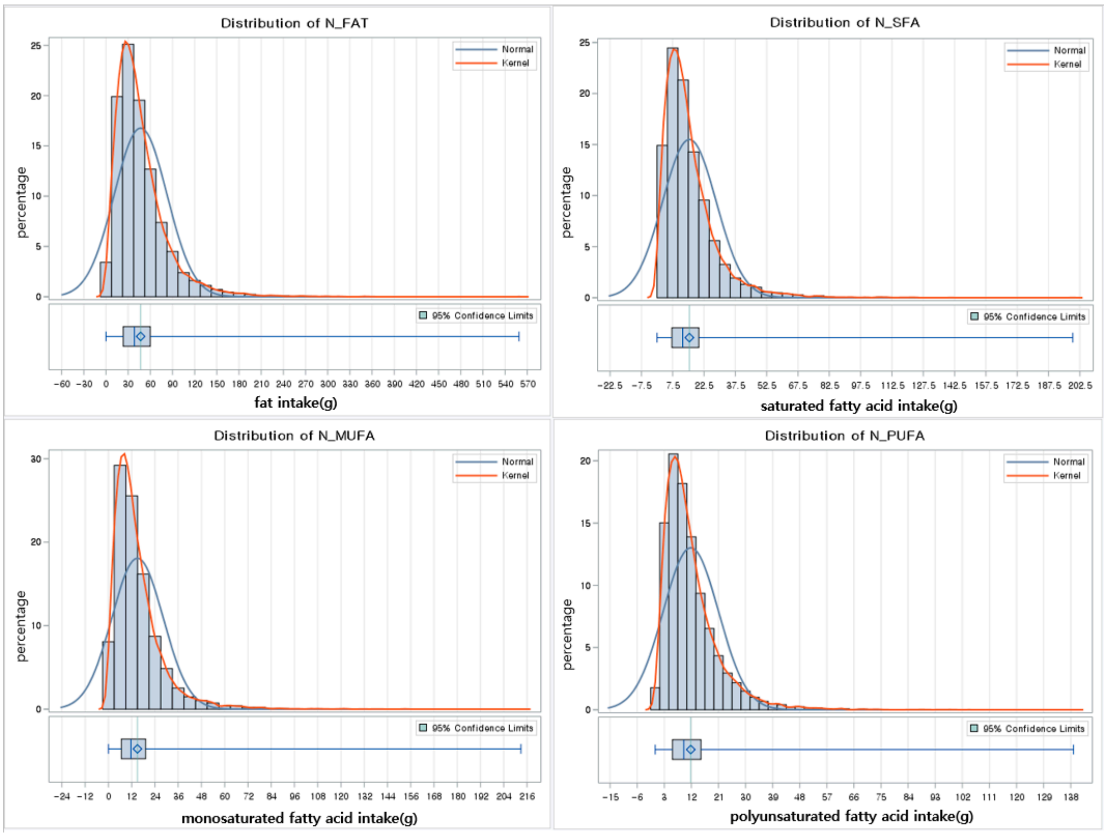
Distribution of N_FAT
Normal
Kernel
95% Confidence Limits
percentage
fat intake(g)
Distribution of N_SFA
Normal
Kernel
95% Confidence Limits
percentage
saturated fatty acid intake(g)
Distribution of N_MUFA
Normal
Kernel
95% Confidence Limits
percentage
monosaturated fatty acid intake(g)
Distribution of N_PUFA
Normal
Kernel
95% Confidence Limits
percentage
polyunsaturated fatty acid intake(g)

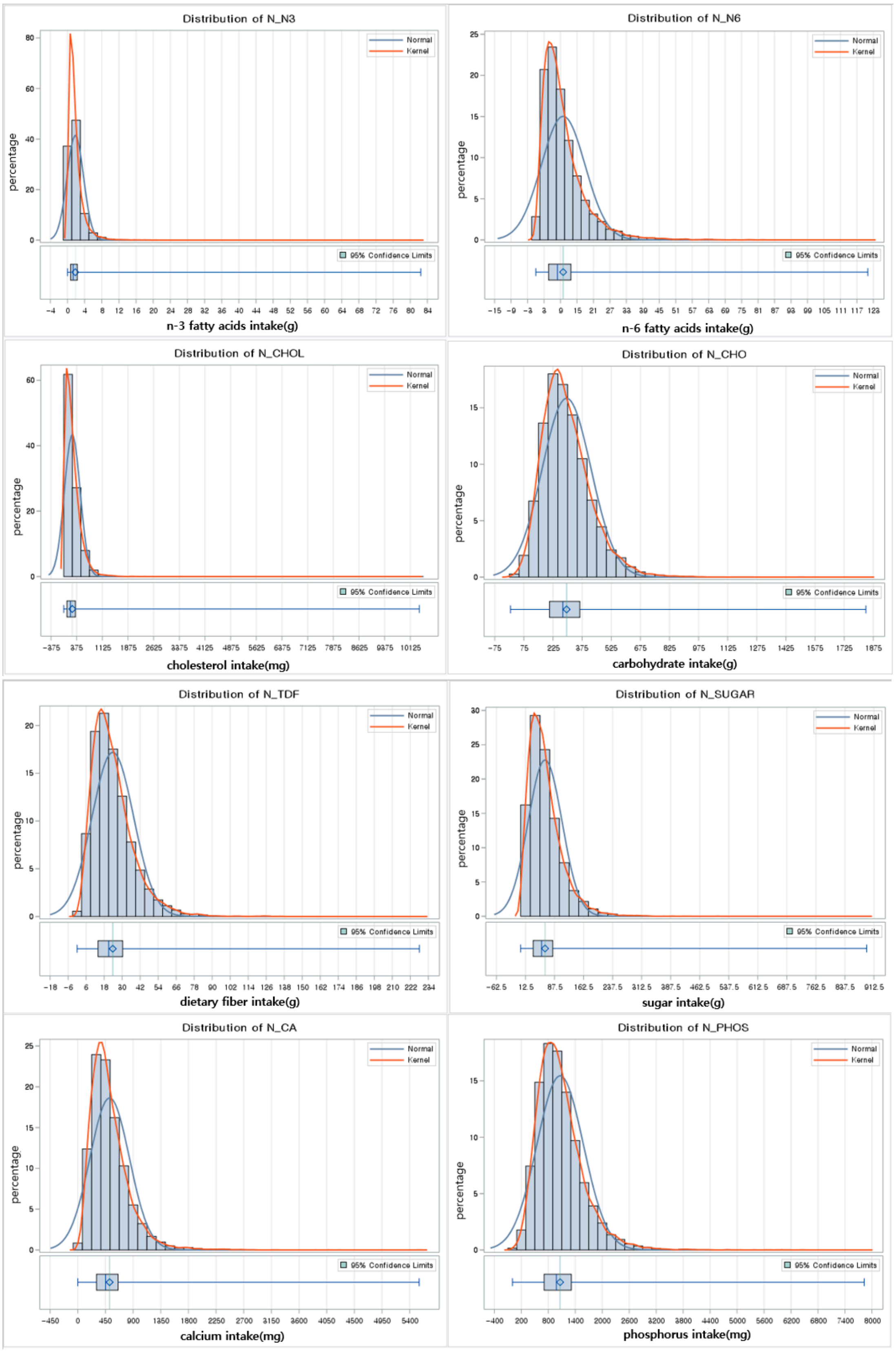

Distribution of N_N3
n-3 fatty acids intake(g)
Distribution of N_N6
n-6 fatty acids intake(g)
Distribution of N_CHOL
cholesterol intake(mg)
Distribution of N_CHO
carbohydrate intake(g)
Distribution of N_TDF
dietary fiber intake(g)
Distribution of N_SUGAR
sugar intake(g)
Distribution of N_CA
calcium intake(mg)
Distribution of N_PHOS
phosphorus intake(mg)
percentage
Normal
Kernel
95% Confidence Limits

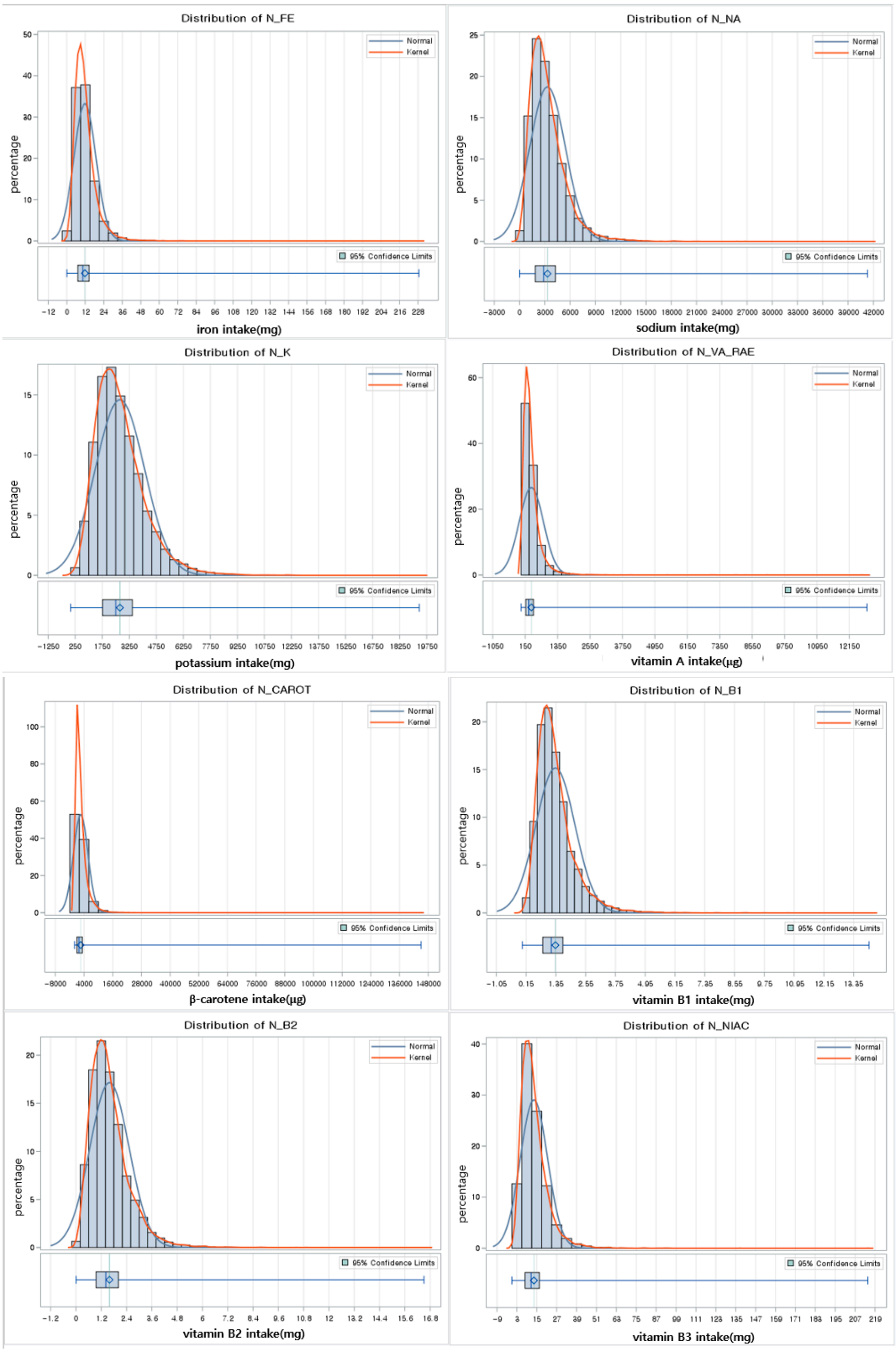

Distribution of N_FE
iron intake(mg)
Distribution of N_NA
sodium intake(mg)
Distribution of N_K
potassium intake(mg)
Distribution of N_VA_RAE
vitamin A intake(μg)
Distribution of N_CAROT
β-carotene intake(μg)
Distribution of N_B1
vitamin B1 intake(mg)
Distribution of N_B2
vitamin B2 intake(mg)
Distribution of N_NIAC
vitamin B3 intake(mg)
percentage
Normal
Kernel
95% Confidence Limits

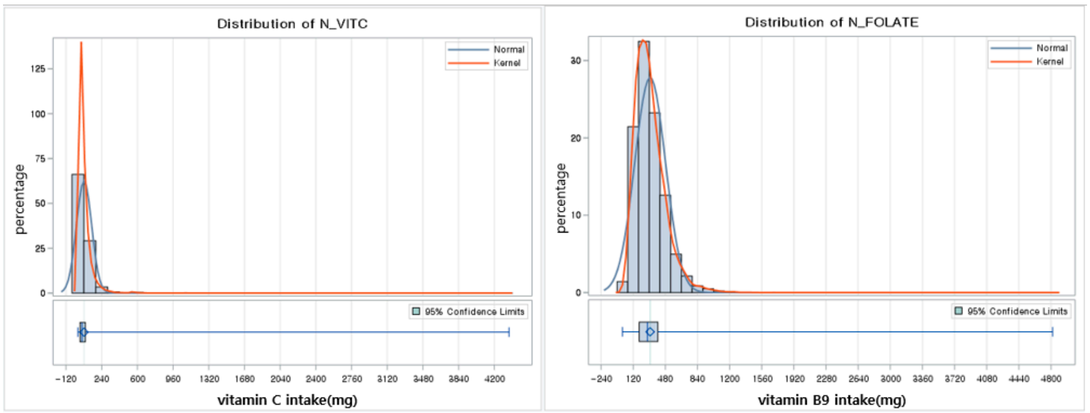

Figure S2. The distribution of 25 nutrient variables.

Table S1. Kolmogorov-Smirnov test performed to confirm the distribution of our data.

**Nutrition**	*P*-value
Water	0.18586
Protein	0.20624
Fat	0.27747
Saturated fatty acids	0.27640
Monounsaturated fatty acids	0.27825
Polyunsaturated fatty acids	0.21894
Omega-3 fatty acid	0.13337
Omega-6 fatty acid	0.23348
Cholesterol	0.24899
Carbohydrates	0.07303
Dietary fiber	0.05113
Sugar	0.16779
Calcium	0.12651
Phosphorus	0.17320
Iron	0.10938
Sodium	0.15738
Potassium	0.12478
Vitamin A	0.13777
Carotene	0.09626
Vitamin B1	0.14638
Vitamin B2	0.21669
Vitamin B3	0.23220
Vitamin B9	0.08047
Vitamin C	0.08528

Table S2. The results of Student *t*-test and Cohen's *d* for study population.

Variables	*P*-value		Cohen's *d*	
	**Male**	**Female**	**Male**	**Female**
**Nutrient intake**				
Food intake	*p*<.001	*p*<.001	0.23	0.22
Energy	0.0002	*p*<.001	0.17	0.2
Water	*p*<.001	*p*<.001	0.21	0.21
Protein	*p*<.001	*p*<.001	0.18	0.22
Fat	0.0643	*p*<.001	0.08	0.18
Saturated fatty acids	0.0884	*p*<.001	0.08	0.16
Monounsaturated fatty acids	0.0383	*p*<.001	0.09	0.18
Polyunsaturated fatty acids	0.2222	*p*<.001	0.06	0.13
Omega-3 fatty acid	0.3877	0.0805	0	0.09
Omega-6 fatty acid	0.2528	*p*<.001	0.05	0.13
Cholesterol	0.0129	0.0009	0.12	0.13
Carbohydrates	0.0137	0.0001	0.11	0.15
Dietary fiber	0.0212	*p*<.001	0.09	0.15
Sugar	0.0135	*p*<.001	0.11	0.17
Calcium	0.0043	0.0236	0.13	0.08
Phosphorus	0.0008	*p*<.001	0.16	0.18
Iron	0.0128	0.0007	0.11	0.12
Sodium	0.0145	0.0015	0.11	0.13
Potassium	0.0006	*p*<.001	0.16	0.19
Vitamin A	*p*<.001	*p*<.001	0.16	0.13
Carotene	0.0210	0.0006	0.11	0.13
Vitamin B1	0.0002	0.0013	0.18	0.13
Vitamin B2	0.0007	*p*<.001	0.24	0.27
Vitamin B3	*p*<.001	*p*<.001	0.16	0.18
Vitamin B9	*p*<.001	*p*<.001	0.12	0.14
Vitamin C	0.0185	0.0500	0.12	0.07

* *P*-value < 0.05

** *P*-value < 0.01

Table S3. The results of univariate simple logistic regression on nutrient intake for cataract among male and female.

Variable		Male Odds Ratio (95% CI)	Female Odds Ratio (95% CI)
Water	Low - mid	1.110 (0.883-1.395)	**1.500 (1.238-1.818)** **
	High - mid	**0.639 (0.501-0.816)** **	0.853 (0.692-1.052)
Protein	Low - mid	1.178 (0.944-1.469)	**1.344 (1.112-1.624)** **
	High - mid	**0.716 (0.535-0.957)** **	**0.733 (0.580-0.926)**
Fat	Low - mid	1.168 (0.950-1.435)	1.207 (1.000-1.457)
	High - mid	0.817 (0.591-1.130)	**0.657 (0.491-0.879)** **
Saturated fatty acids	Low - mid	1.239 (0.995-1.544)	**1.266 (1.062-1.508)** **
	High - mid	1.011 (0.739-1.384)	**0.711 (0.506-0.999)**
Monounsaturated fatty acids	Low - mid	1.261 (1.023-1.554)	1.153 (0.962-1.382)
	High - mid	0.853 (0.568-1.282)	**0.651 (0.493-0.860)** **
Polyunsaturated fatty acids	Low - mid	1.044 (0.846-1.290)	**1.428 (1.195-1.705)** **
	High - mid	0.940 (0.703-1.256)	0.873 (0.689-1.105)
Omega-3 fatty acid	Low - mid	1.118 (0.887-1.409)	**1.325 (1.086-1.617)** **
	High - mid	0.975 (0.773-1.229)	0.912 (0.740-1.123)
Omega-6 fatty acid	Low - mid	1.120 (0.885-1.371)	**1.217 (1.012-1.462)** **
	High - mid	1.008 (0.752-1.350)	**0.743 (0.583-0.946)** **
Cholesterol	Low - mid	1.246 (1.004-1.547)	**1.382 (1.150-1662)** **
	High - mid	0.946 (0.707-1.265)	1.015 (0.793-1.299)
Carbohydrates	Low - mid	1.015 (0.790-1.304)	**1.278 (1.014-1.611)** **
	High - mid	0.773 (0.614-0.974)	**0.786 (0.649-0.951)** **
Dietary fiber	Low - mid	0.920 (0.691-1.227)	**1.361 (1.089-1.701)** **
	High - mid	**0.754 (0.604-0.941)** *	**0.750 (0.621-0.905)** **
Sugar	Low - mid	1.017 (0.799-1.294)	**1.537 (1.274-1.854)** **
	High - mid	0.747 (0.571-0.978)	0.836 (0.672-1.040)
Calcium	Low - mid	1.200 (0.937-1.538)	**1.244 (1.018-1.519)** **
	High - mid	**0.838 (0.657-1.068)** *	**0.819 (0.677-0.992)** **
Phosphorus	Low - mid	1.089 (0.871-1.361)	**1.403 (1.157-1.701)** **
	High - mid	0.787 (0.610-1.015)	0.834 (0.683-1.019)
Iron	Low - mid	0.975 (0.749-1.269)	**1.333 (1.074-1.654)** **
	High - mid	0.774 (0.606-0.987)	0.869 (0.722-1.046)
Sodium	Low - mid	1.050 (0.849-1.298)	1.190 (0.982-1.442)
	High - mid	0.860 (0.666-1.109)	0.905 (0.738-1.108)
Potassium	Low - mid	0.985 (0.770-1.260)	1.444 (1.167-1.785)
	High - mid	**0.737 (0.590-0.920)** *	**0.770 (0.638-0.928)** **
Vitamin A	Low - mid	1.131 (0.880-1.453)	**1.625 (1.338-1.973)** **
	High - mid	0.852 (0.644-1.127)	1.029 (0.812-1.303)
Carotene	Low - mid	0.909 (0.706-1.171)	**1.329 (1.088-1.623)** **
	High - mid	0.761 (0.602-0.963)	0.830 (0.681-1.012)
Vitamin B1	Low - mid	1.126 (0.895-1.418)	**1.312 (1.061-1.621)** **
	High - mid	**0.640 (0.494-0.829)** **	0.868 (0.709-1.063)
Vitamin B2	Low - mid	1.213 (0.968-1.520)	**1.593 (1.320-1.922)** **
	High - mid	0.806 (0.620-1.047)	0.963 (0.779-1.191)
Vitamin B3	Low - mid	1.278 (1.032-1.582)	**1.571 (1.300-1.899)** **
	High - mid	**0.694 (0.529-0.912)** **	0.845 (0.669-1.068)
Vitamin B9	Low - mid	0.962 (0.756-1.225)	**1.336 (1.093-1.634)** **
	High - mid	0.786 (0.617-1.000)	0.844 (0.699-1.019)
Vitamin C	Low - mid	1.140 (0.916-1.419)	**1.314 (1.073-1.609)** **
	High - mid	0.824 (0.649-1.046)	0.917 (0.750-1.122)
Food intake	Low - mid	1.262 (1.011-1.575)	**1.476 (1.217-1.790)** **
	High - mid	**0.699 (0.536-0.911)** **	0.815 (0.656-1.013)
Energy	Low - mid	1.085 (0.866-1.359)	1.329 (1.082-1.633)
	High - mid	**0.595 (0.454-0.780)** **	**0.719 (0.585-0.883)** **
Education	Less than middle school-higher than high school	**1.248 (1.033-1.508)** *	**1.690 (1.3868-2.086)** **

National basic livelihood	Non-beneficiaries - beneficiaries	1.275 (0.899-1.809)	**2.038 (1.563-2.658)** **
Marital status	Single - married	1.351 (0.558-3.274)	1.339 (0.560-3.206)
Age	60s – 70s	**0.266 (0.216-0.327)** **	**0.222 (0.187-0.265)** **
Obesity	Absence - presence	1.015 (0.808-1.274)	1.138 (0.926-1.398)
Hypertension	Absence – presence	**0.650 (0.528-0.801)** **	**0.531 (0.444-0.635)** **
Diabetes	Absence - presence	**0.591 (0.480-0.727)** **	**0.568 (0.480-0.671)** **
Asthma	Absence - presence	**0.351 (0.198-0.621)** **	**0.598 (0.412-0.867)** **
Sinusitis	Absence - presence	0.643 (0.413-1.001)	0.725 (0.505-1.041)
Allergic rhinitis	Absence - presence	0.898 (0.610-1.321)	1.235 (0.898-1.699)
Hyperlipidemia	Absence - presence	**0.768 (0.624-0.945)** *	0.920 (0.775-1.092)
Heart failure	Absence - presence	**0.293 (0.112-0.767)** *	0.546 (0.203-1.463)
Physical activity	No - Yes	1.209 (0.982-1.488)	**1.374 (1.135-1.662)** **
Smoke	Non-smoker – smoker	**1.281 (1.002-1.638)** *	0.900 (0.533-1.521)
Heavy drinking	Non-Heavy drinking - Heavy drinking	**2.417 (1.679-3.478)** **	1.630 (0.744-3.572)

* $P< 0.05$

** $P<0.01$

Table S4. The VIF value of the independent variables used in univariate multiple logistic regression in male and female.

Nutrition	Male (VIF value)	Female (VIF value)
Water	1.94	2.05
Protein	2.84	4.04
Fat	-	6.76
Saturated fatty acids	-	3.74
Monounsaturated fatty acids	1.75	4.21
Polyunsaturated fatty acids	-	8.96
Omega-3 fatty acid	-	2.35
Omega-6 fatty acid	-	7.42
Cholesterol	-	2.14
Carbohydrates	-	2.02
Dietary fiber	2.00	2.70
Sugar	-	1.77
Calcium	1.88	2.28
Phosphorus	-	4.97
Iron	-	2.47
Potassium	3.10	3.62
Vitamin A	-	2.53
Carotene	-	2.07
Vitamin B1	1.99	2.17
Vitamin B2	2.63	2.91
Vitamin B3	2.47	2.57
Vitamin B9	-	2.60
Vitamin C	-	1.63
